# The corporate capture of the nutrition profession in the USA: the case of the Academy of Nutrition and Dietetics

**DOI:** 10.1017/S1368980022001835

**Published:** 2022-12

**Authors:** Angela Carriedo, Ilana Pinsky, Eric Crosbie, Gary Ruskin, Melissa Mialon

**Affiliations:** 1 World Public Health Nutrition Association, 46 Hoddern Av, Peacehaven, BN10 7PH, UK; 2 Department of Health, University of Bath, Bath, UK; 3 Urban Food Policy Institute, Graduate School of Public Health and Health Policy, City University of New York, New York, USA; 4 School of Public Health, University of Nevada Reno, Reno, NV, USA; 5 Ozmen Institute for Global Studies, University of Nevada Reno, Reno, NV, USA; 6 US Right to Know, Oakland, CA, USA; 7 Trinity College Dublin, Dublin, Ireland

**Keywords:** Corporate influence, Academy of Nutrition and Dietetics, Nutrition profession, Health policy

## Abstract

**Objective:**

The involvement of unhealthy commodity corporations in health policy and research has been identified as an important commercial determinant contributing to the rise of non-communicable diseases. In the USA, health professional associations have been subject to corporate influence. This study explores the interactions between corporations and the Academy of Nutrition and Dietetics (AND), and their implications for the profession in the USA and globally.

**Design:**

We conducted an inductive analysis of documents (2014–2020) obtained through freedom of information requests, to assess key AND actors’ dealings with food, pharmaceutical and agribusiness corporations. We also triangulated this information with publicly available data.

**Setting:**

The USA.

**Participants:**

Not applicable.

**Results:**

The AND, AND Foundation (ANDF) and its key leaders have ongoing interactions with corporations. These include AND’s leaders holding key positions in multinational food, pharmaceutical or agribusiness corporations, and AND accepting corporate financial contributions. We found the AND has invested funds in corporations such as Nestlé, PepsiCo and pharmaceutical companies, has discussed internal policies to fit industry needs and has had public positions favouring corporations.

**Conclusion:**

The documents reveal a symbiotic relationship between the AND, its Foundation and corporations. Corporations assist the AND and ANDF with financial contributions. AND acts as a pro-industry voice in some policy venues, and with public positions that clash with AND’s mission to improve health globally.

The rising global burden of non-communicable diseases has for decades been addressed by downstream efforts that focus on improving individual behaviours^(1)^. However, recently upstream efforts focused on societal and environmental changes have led to important population-level approaches and policies implemented in several countries to improve non-communicable diseases, including obesity and diabetes^(2,3)^. An important barrier to these approaches is the commercial determinants of health^(4,5)^. These are actions, processes and ways in which commercial actors such as unhealthy commodity corporations (tobacco, alcohol and ultra-processed food and drink) influence health policy making and, in general, influence the environment to protect their interests^(6)^.

There is extensive literature that shows how unhealthy commodity corporations are involved in setting health policy and research agendas globally^(7,8)^. In particular, they use instrumental (action-based) and discursive (argument-based) strategies to influence science and policy surrounding public health efforts to protect well-being and healthy environments^(6,9)^. Furthermore, corporations lobby and litigate against health policies and capture science by recruiting and hiring scientists to influence public discourse and position corporate interests in the public agenda^(8,10,11)^. One key strategy is to capture health professionals and health institutions as a vehicle to achieve its interests more broadly in the global health agenda.

In the USA, one of the most important professional health associations is the Academy of Nutrition and Dietetics (AND)^(12–14)^. The AND’s relationship with the food and beverage industry has been described elsewhere^(15,16)^. Founded in 1917 as the American Dietetic Association, the AND is the largest US-based organisation comprised of food and nutritional professionals, with approximately 100 000 dietitians and nutrition practitioners and students^(17)^. It is established as a 501(c)(6) trade association and certifies dieticians and nutrition practitioners in the USA and abroad^(17,18)^. The AND’s stated mission is ‘to accelerate improvements in global health and well-being through food and nutrition’. AND acts as a reference for dietetics curricula accreditation and as an authority in US food policy making^(19)^. For instance, the Academy has been influential in the process of setting US Dietary Guidelines, which are then taken into consideration all over the world in order to develop vital nutrition policy decisions^(14,20)^. The AND also provides ‘expert testimony’ including ‘comments and position statements for federal and state regulations on critical food and nutrition issues’^(17)^. The ‘philanthropic arm’ of the AND is the AND Foundation (ANDF), established as a 501(c)(3) charitable organisation. The ANDF does not receive member dues and relies on donations. It focuses on scholarships, awards, food and nutrition research and public education^(21)^. The AND and the ANDF report jointly their annual activities and achievements, without a clear distinction between each another. They also share staff, including the chief executive officer and chief of operations^(21)^.

The AND has been repeatedly criticised for its close ties to food and beverage corporations, including Coca-Cola, PepsiCo and General Mills, which may undermine ‘the integrity of the professionals most responsible for educating Americans about healthy eating’^(22)^. Two years after the publication of a critical report about AND’s relationship with food corporations in 2013, the ANDF announced a partnership with the food company Kraft^(16)^. This collaboration, which was seen as an endorsement of some of Kraft’s products as ‘healthy’ options to include in children’s menus at schools, caused further outrage among AND’s members, public health experts and the general public^(15,20,23)^.

Although the AND’s relationship with the food and beverage industry has been described before^(15,16,20)^, little is known about its relationship with other unhealthy commodity industries as well as the dynamics and evolution of such relationships. This study is the first to obtain and review AND’s internal communications and interactions between the AND and the food and beverage, pharmaceutical and agribusiness industries. We explore how these interactions evolved over time^(15,16,20)^ and how they influence the politics and decision-making of an influential professional health association, by analysing documents obtained through freedom of information (FOI) requests, filed by US Right to Know (USRTK).

## Materials and methods

The USRTK, an investigative public health group, obtained internal AND communications through FOI requests. On 21 December 2017, USRTK filed a Georgia Open Records Act request to the Burke County Public Schools for email correspondence to or from Donna Martin, an AND leader who served in the AND for more than 10 years. The request asked for any mention in those records of key companies in the US food market: Splenda, Heartland Food Products Group, Tate & Lyle, Abbott Nutrition, Ingredion, Pepsi, Coca-Cola, as well as the American Beverage Association, based on previous publications pointing out some corporate relationships. Burke County Public Schools provided a total of 28 204 pages in response. USRTK sent two more FOI requests in 2019 and 2020. Burke County Public Schools responded with 53 684 pages and attachments, and then 27 more emails dated between January 2018 and March 2019. USRTK’s requests were for records after January 2013, the date when the first report criticising AND’s corporate ties was published, until September 2020^(22)^.

We reviewed the documents and coded them inductively between September and November 2020. Two authors reviewed an additional 10 % of the documents to improve coding validity and reliability. The team met every 2 weeks to discuss coding and findings for consensus and reflection about the process and findings.

Additionally, to identify gaps and clarify key events described in the FOI documents, we triangulated the findings with an online search for documents in English. We searched the AND and ANDF websites, used the Wayback Machine (an online tool that captures historical versions of websites) and used Google Scholar and Google to collect further information about the AND and its Foundation, using a combination of key search terms including ‘AND’, ‘ANDF’, ‘Eat Right’, ‘sponsorship’, ‘corporate’, ‘mission’ and ‘Sponsorship task force’ as well as key names of leaders identified as relevant to our research objectives (online Supplementary material 1).

First, we mapped the actors and the timeline of events relevant to our study. We then conducted an inductive analysis. Any information from either the FOI documents or public AND documents (websites) related to corporate influence was captured.

The key information we identified in our data was (a) interactions between key leaders of the AND/ANDF and corporations, (b) corporate financial contributions to the AND/ANDF shown in Internal Revenue Service form 990 Schedule B tax returns or (c) the AND/ANDF internal policies and public positions since the Kraft partnership was announced in 2015 that generated public attention and outraged members^(15)^.

The key emerging themes from that information were (a) use of revolving doors between AND’s board (BOD) of directors with corporate interests and (b) investments of AND in corporations and corporations funding AND/ANDF and their events. We also noted that corporations: (a) financed early career nutritionists and their research; (b) interfered with AND position papers on key nutrition related topics and themes and (c) led to the shaping of internal policies that benefit corporate partners.

We present a narrative review of our results in the form of mechanisms we identified in which corporations might exert their influence in and through the AND/ANDF. We also identify the ways in which the AND/ANDF have benefited from these corporate interactions over time. For the purpose of this paper, the term ‘influence’ (or ‘power’) is defined ‘as an actor’s ability to induce or influence another actor to carry out his (/her) directives or any norms he (/she) supports’^(24–26)^. We use ‘the Academy’ to refer to both the AND and ANDF, and we use ‘corporations’ or ‘industry’ to refer to unhealthy commodity corporations, including the food and beverage, pharmaceutical and agribusiness industries.

## Results

Following a report published in 2013 denouncing AND’s close relationships with the food industry, the AND established a Sponsorship Advisory Task Force (SATF) to improve its Corporate Sponsorship Guidelines^(27)^. In 2015, when AND’s partnership with Kraft was disclosed and criticised by the public, the AND/ANDF BOD dropped the deal. However, the documents gathered through FOI show they privately continued to engage with corporations by: (i) investing AND funds in shares of Nestlé, PepsiCo and several pharmaceutical company stocks; (ii) accepting corporate contributions without disclosing their size, (iii) allowing BOD members to work for or consult for companies with interests that conflict with the mission of the AND, (iv) discussing internal policies within the BOD to fit industry needs, ignoring the work of the SATF, (v) allowing corporations to support AND’s members research and (vi) releasing public positions favouring corporations.

**Fig. 1:**
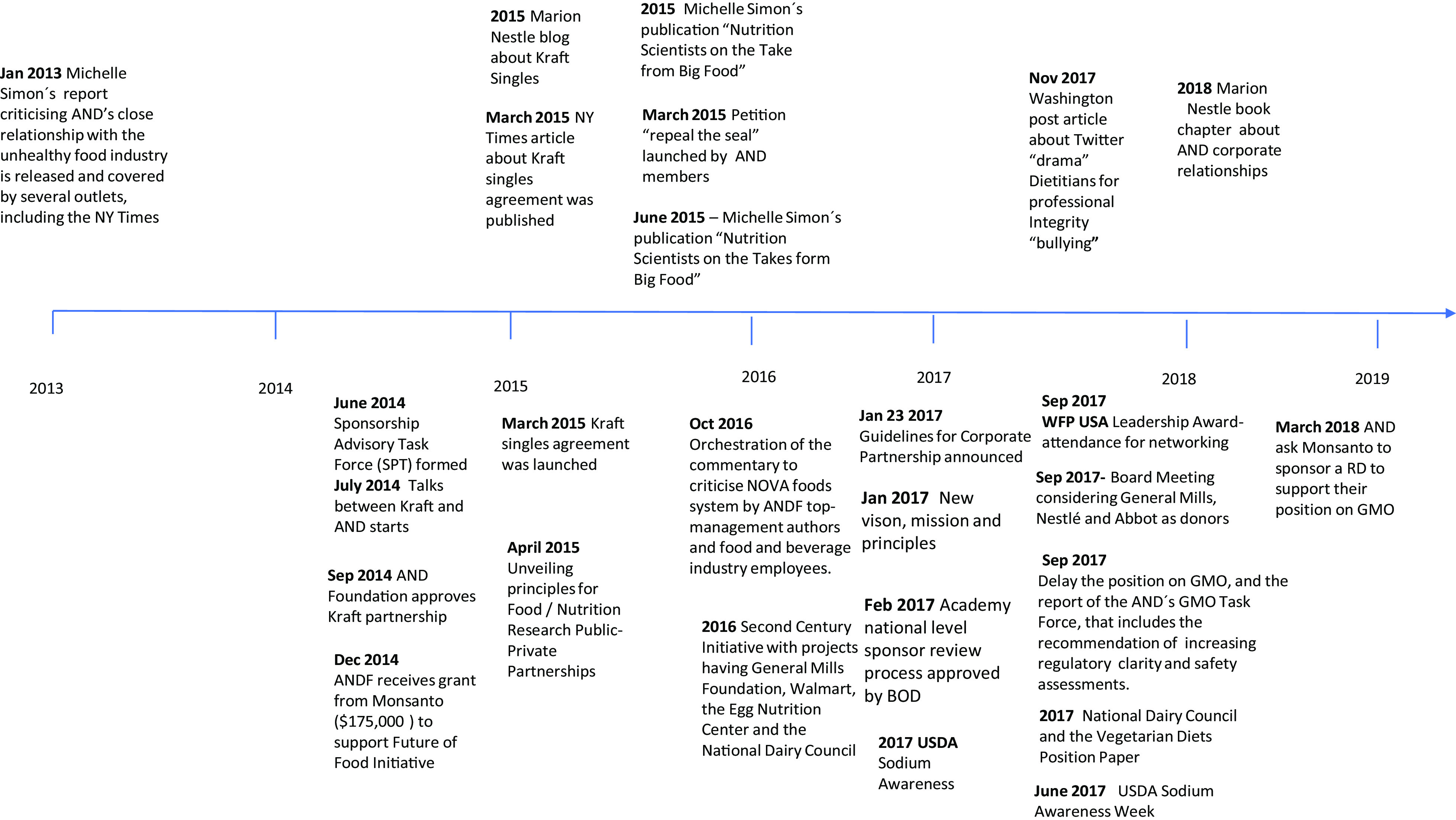
Timeline of Academy of Nutrition and Dietetics (AND) and its critics key events around corporate interactions

In 2017, after ‘unprecedented pressure from members and the public on the Academy’s sponsorship relationships’, the BOD presented to its members a new mission, vision and principles, accompanied by the new ‘*Guidelines for Corporate Sponsors’* and ‘*Guiding Principles of the Academy’s Corporate Sponsorship Program*’^(28,29)^. Nonetheless, AND continued to accept corporate funding and engage in corporate partnerships such as a fundraising effort for their Second Century initiative and for the sponsorships for students and dietitians^(30,31)^. Figure 1 presents a timeline of key actions we identified regarding AND/ANDF interactions with corporations.

### Academy of Nutrition and Dietetics/Academy of Nutrition and Dietetics Foundation governing bodies’ interactions with corporations

**Fig. 2:**
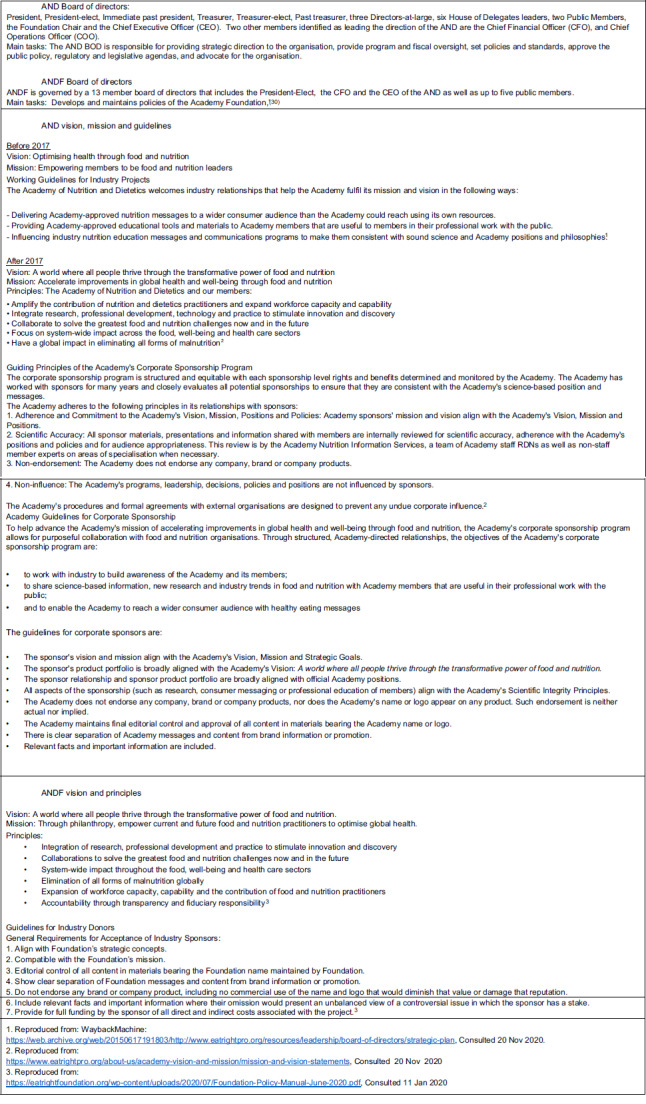
Academy of Nutrition and Dietetics (AND) and AND Foundation (ANDF) governing bodies, vision, mission and principles (from 2017)

The AND’s BOD and the ANDF’s BOD are comprised of nineteen individuals and thirteen members, respectively. Details on the mission and vision of each are described in Fig. 2. Major internal policies are approved by the AND’s and ANDF’s directors and are reviewed and adopted annually^(21)^.

After mapping some of the key AND and ANDF’s BOD, we identified several key members that have had close relationships with corporations throughout the years we mapped (2009–2020). Online Supplementary material 1 outlines a non-exhaustive list of these key AND members.

Donna Martin, an influential AND member who has encouraged corporate connections, has served in several positions for the Academy: as treasurer (2013–2015), president-elect (2016–2017) and president (2017–2018). In 2015, she agreed to endorse Kraft Singles, despite their poor nutritional value. Additionally, when commenting on a CFO’s report about the AND investment portfolio, she mentioned to another Academy’s executive member:

Everything looks good to me. The only flag that I saw was that PepsiCo is one of our top ten stocks (in which AND has invested). I personally like Pepsico and do not have any problems with us owning it, but I wonder if someone will say something about that. Hopefully they will be happy like they should be! I personally would be OK if we owned Coke stock!! (Donna Martin, email, 3^rd^ January 2014)

Another AND director, Milton Stokes, was an employee at Monsanto in 2014, and from 2014 to 2020 he was the Global Lead, Public Affairs and Issues Management at Bayer Crop Science (a subsidiary of Bayer, which now owns Monsanto). Monsanto has donated at least $395 000 to the AND and worked closely with the AND, especially after Stokes joined Monsanto. For instance, in 2015 Monsanto contributed $175 000 for the Foundation’s ‘*Future of Food Initiative*’. The same year, Monsanto established an advisory group with fourteen former AND board members and had several AND/ANDF members as spokespeople ‘to serve on a two-year contractual basis as communication advisors’ (Milton Stokes, 11th December 2014). In 2017, he emailed several members of the AND leadership team mentioning that Monsanto has committed to support the Academy financially, and recruited AND members ‘to raise the visibility of the nutrition and dietetics community with Monsanto’ and arrange ‘a visit for the AND’s scientific officer to learn about Genetic Modified Organism (GMO) use in Nairobi, and Kenya’.

In 2017, as an employee of Monsanto, Stokes contacted two leaders to further collaborate with the AND to align agendas around sustainable diets, mentioning other industry-funded organisations involved:

‘to support convening experts from various disciplines to share the language, metrics and goals for each [sector] and then align on a research agenda that would allow for a more meaningful discussion of the science, including an acknowledgement of trade-offs. This work started already by ILSI’. (Milton Stokes, 11th May 2017)

In September 2017, as member of the BOD, Stokes invited the AND CEO to attend the World Food Program-USA Leadership Awards ceremony to connect with leaders on the topic (sustainable diets) as an attempt to ‘further advance the agenda on sustainable diets’ (15th September 2017), calling it a strategic meeting with government, illustrating the continued close relationships with Monsanto.

### The Academy’s corporate financial contributions and its corporate investments

The AND has maintained financial ties to food, pharmaceutical and agribusiness corporations, despite criticism and the potential reputational risks identified by some ex-Academy members^(32)^. We found three main types of financial ties. First, FOI documents revealed the corporate financial contributions to the AND for the years 2011, and 2013 to 2017 (Table 1). In 2011, the AND received more than US$300 000 from Hershey Co., a chocolate manufacturer, and nearly US$300 000 from the National Dairy Council (NDC), Conagra, Coca-Cola and Aramark, a company providing food services. Abbott, a pharmaceutical company selling infant formula, as well as General Mills and Cargill each donated more than US$100 000 in 2011 and maintained substantial donations from 2013 to 2017. Food and beverage companies such as Nestlé, Coca-Cola and PepsiCo, with the exception of General Mills, reduced their contributions over time. Nevertheless, contributions from companies such as Pharmavite-Nature Made and Abbott increased substantially during this same period. Overall, contributions shrunk by more than US$600 000 in 2015 and by more than US$500 000 in 2016, in respect to previous years.

**Table 1:** Corporate and organisational contributions to the AND 2011–2017 in USD

Contributors	2011	2013	2014	2015	2016	2017	Total
A Cook’s Tour	13 750			11 750			25 500
A2 Milk Company				13 200	13 815		27015
Abbott Healthcare	5000	5000					10 000
Abbott Laboratories	1 87 192	2 03 948	2 73 791	26 523	38 500	94 156	8 24 110
Abbott Laboratories Trading (Shanghai)				28 000			28 000
Abbott Nutrition	1 42 772	3 48 817	2 72 886	2 02 682	2 03 900	75 332	12 46 389
AbbVie Inc.		5000		7500			12 500
Academy of Nutrition and Dietetics Foundation	1 82 309	97 029	1 01 803	65 325	45 863	3 08 932	8 01 261
Agency for Healthcare Research and Quality		96 495	64 142	1 35 858			2 96 495
Agro-Farma	25 750						25 750
Ajinomoto Inc.	29 500	16 000	19 500		18 000		83 000
Alaska Seafood Marketing Institute		15 600					15 600
Alcresta Inc.		5000		27 500	7500		40 000
Allergan Inc.					7500		7500
American Beverage Association				10 000			10 000
American Egg Board			10 000				10 000
American Pistachio Growers			10 000				10 000
Aptalis Pharmaceuticals		7500					7500
Aramark Corporation	2 78 051	15 000					2 93 051
Arla Foods					20 000		20 000
Atkins Nutritionals Inc.				5000			5000
Australis Barramundi					10 000		10000
Bariatric Fusion LLC						26 870	26870
Baxter Healthcare Corporation			43 000		25 000		68 000
Beadle consulting LLC DBA Salt & Company				17 500			17500
Becton Dickinson & Co	36 000						36 000
Beijing E-Jane Healthcare Management					37 000		37000
Beneo Inc.				50 000	92 833		142833
Biosan Laboratories Inc.	5000						5000
BioVittoria	5000						5000
BiPro USA			6500				6500
BMIQ	15 000						15 000
BodyMedia	3 000						3 000
Boehringer Ingelheim Pharmaceuticals				20 000			20 000
Bumble Bee Foods				10 000			10 000
Butter Buds	8 736						8 736
California Beef Council			7 500				7 500
California Table Grape Commission			22 000				22 000
California Walnut Commission	6 500			8 500			15 000
Calorie Control Council	5 500						5 500
Campbell Soup Company	16 700	16 600	22 350	12 500	12 400		80 550
Canola Council of Canada	18 000	15 000	21 250	14 500		7 000	75 750
Cargill Inc. – Truvia	1 19 280	35 150					1 54 430
Case Western Reserve University				5 650			5 650
CDR Transfer	6 000						6 000
Cell Science Systems Corp	5 000						5 000
Chobani					10 000		10 000
Cinsulin						6 500	6 500
Clif Bar & Company		7 060	7 500	7 500	21 500	14 200	57 760
CMGRP Inc.	32 500	48 400	40 126				1 21 026
Coca-Cola Company	2 86 025	1 78 102	13 450				4 77 577
Computrition Inc.		5 500					5 500
Conagra Foods		12 500					12 500
Conagra Inc.	2 90 453	3 30 211	4 55 894	3 04 167	33 333		14 14 058
Corn Refiners Association	18 638			5 000			23 638
Covidien	8 750	10 000	30 000				48 750
Coyne Public Relations							
Cropp Cooperative Inc.		5 000	5 000	5 000			15 000
Culinary Institute of America	5 907						5 907
Dairy Management Inc.			20 000		10 000		30 000
Daisy Brand		9 000	5 000				14 000
Dannon				25 000			25 000
DayTwo						13 000	13 000
Del Monte Corporation		16 600					16 600
DNA Dreamfields Company, LLC	6 500	10 200					16 700
Domino Foods, Inc.			10 100	5 000		9 000	24 100
DPG 31 DHCC			5 500				5 500
DuPont Pioneer						9 000	9 000
Eating Recovery Center			10 000				10 000
Edelman Public Relations Worldwide	30 000	5 000	6 500				41 500
Egg Nutrition Center		5 000	10 087	15 230			30 317
Elanco Global Communication				25 000			25 000
Eli Lilly and Company	63 785	36 000	36 000	36 000	36 000	18 000	2 25 785
Emerson Ecologics, Inc.					8 440		8 440
Entrinsic Health Solutions						7 500	7 500
Evans Hardy + Young Inc.		19 000	14 000	15 130		27 700	75 830
First Food Marketing					6 000		6 000
Fleishman–Hillard	10 000	40 000	9 000	5 500		16 000	80 500
FoodMinds, LLC	13 663	46 150	46 500	31 920	58 863	51 900	2 48 996
Fresenius Kabi USA				10 250			10 250
Fruit Street Health PBC				39 050			39 050
Gaia Herbs Inc.			10 651	6 641	11 358		28 650
Gatorade Company		10 000	82 679		5 000	10 000	1 07 679
General Mills	1 23 560	86 711	57 462	14 000	14 000	14 000	3 09 733
Ginger Network						5 500	5 500
GlaxoSmithKline		10 000					10 000
Global Organisation for EPA and DHA						10 000	10 000
Glutamate Association			15 000				15 000
GMRI Inc.			5 000				5 000
GW Hoffman Marketing & Communication c/o Dannon				10 000		10 000	
Hass Avocado Board		15 600	38 500	31 550	21 200		1 06 850
Health and Nutrition Technology Inc.				7 500			7 500
Heartland Food Products Group				22 150	33 200	19 500	74 850
Herbalife			5 000				5 000
Hershey Co.	3 22 627	45 405					3 68 032
Hydration Pharmaceuticals Trust					21 200		21 200
ILSI North America					5 000		5 000
Integrated Marketing Group	11 000						11 000
Integrative Therapeutics Inc.					5 100		5 100
International Food Information Council Foundation			7 119			7 119	
Jamba Juice		13 600					13 600
Janssen Pharmaceuticals	7 000		5 500	24 500	7 500	18 000	62 500
Johnson & Johnson		9 000	77 500				86 500
K & M Communications					5 000		5 000
Kate Farms						5 000	5 000
Kellogg USA Inc.	88 680	1 01 247	56 545	26 800			2 73 272
Ketchum Inc.	12 000	9 800	17 000	17 500	61 000		1 17 300
Kind Management Inc.				7 500			7 500
La Sutherland Group		5 000					5 000
LifeScan Inc./Johnson & Johnson					5 000	10 000	15 000
Lifeway Foods Inc.					5 000		5 000
Linhart Public Relations				5 000			5 000
Lundberg Family Farms	5 000						5 000
Masterfoods USA	75 780						75 780
McCormick & Co		70 894	31 086				1 01 980
McNeil Nutritionals Inc.	52 670		14 000				66 670
McNeil/Johnson & Johnson	25 500						25 500
Mead Johnson Nutrition	31 500	37 200	30 700	29 700	25 500	40 500	1 95 100
Mead Johnson Nutritionals	11 764						11 764
Medical Nutrition USA			5 000				5 000
Medifast Inc.	5 000		5 000	5 000			15 000
Medtrition						5 000	5 000
Medtronic				5 000			5 000
Metagenics				7 600	5 500		13 100
Modern PR					9 000		9 000
Monsanto Company	5 000		24 000	5 000	78 000	6 000	1 18 000
MSLGroup	5 000		15 204	21 500	8 000	18 500	68 204
Mullen		10 000	5 000				15 000
National Cattlemen’s Beef Association			10 000		25 000	12 300	47 300
National Collegiate Athletic Association		15 188	36 850				52 038
National Dairy Council	2 95 055	3 01 910	2 78 452	2 93 518	3 22 977	5 000	14 96 912
National Kidney Foundation-Nutrition Guidelines for Chronic Kidney Disease	14 000	7 500	18 000	39 500			
National Osteoporosis Foundation					12 400		12 400
National Peanut Board			5 000				5 000
National Processed Raspberry Council		23 000	15 000		17 500		55 500
National Starch	6 000						6 000
National Watermelon Promotion Board					15 000		15 000
Nature Made				12 500			12 500
Nebraska Beef Council			7 500				7 500
Nestle	33 250						33 250
Nestle Frozen Food		5 250					5 250
Nestle Healthcare Nutrition				30 750			30 750
Nestle USA	5 000						5 000
Nestle USA Food	45 000	36 485	31 750	15 500	13 750	5 750	1 48 235
New Balance Foundation				5 000			5 000
North Carolina Sweet Potato Commission			15 000				15 000
Novo Nordisk Inc.		96 667	44 997	34 587			1 76 251
Nutricia Advanced Medical Nutrition	9 500						9 500
Nutricia North America				11 000		5 000	16 000
Nutritional Medicinals LLC				5 500		6 550	12 050
Opal Food and Body				5 000			5 000
Orgain Inc.	5 000					17 750	22 750
Organic Valley Family of Farms	5 000						5 000
Ottawa Hospital Research Institute		10 000					10 000
Outloud				16 250	29 000		45 250
PAR Pharmaceutical Inc.		20 000					20 000
Paramount Farms Inc.	20 000						20 000
Paula Deen Enterprises LLC	50 000						50 000
PepsiCo	68 796	1 17 546	1 97 970	1 02 023			4 86 335
Pew Charitable Trusts						52 524	52 524
Pharmavite-Nature Made	87 480	13 600	21 600	50 000	49 000		2 21 680
Pharmavite-Soyjoy		75 369					75 369
Pollock Communications	85 250	47 500	19 925	29 000	12 000	15 800	2 09 475
POM Wonderful					25 000		25 000
Porter Novelli	5 100	23 500		8 750	11 000		48 350
Premier Nutrition Corp					26 200		26 200
Publications International	13 000						13 000
Publicis Inc.	11 500						11 500
Pure Encapsulation					10 250	12 800	23 050
Raymond Terri J.					10 000		10 000
Red Bull North America			10 000	6 000		10 000	26 000
Roche Diagnostics		36 000	18 000	18 000	18 000		90 000
Rxmosaic					5 000		5 000
Safeway Inc.	16 700	13 600					30 300
Sage Leaf Communications LLC						10 000	10 000
San Miguel Produce Inc.			8 000				8 000
Sanofi Aventis US Inc.				21 500	18 000		39 500
Saskatchewan Pulse Growers				14 000	22 650	48 665	85 315
Sharecare Inc.	33 334						33 334
Shasta Sales Inc.	1 000						1 000
Solae	6 500	6 000	10 200				22 700
Soyfoods Association of North America		11 250					11 250
SpectraCell Laboratories Inc.		10 000					10 000
Stafford Communications Group	6 000						6 000
Sterling-Rice Group, Inc.		12 000	6 000	9 300			27 300
Stonyfield Farm		5 000					5 000
Sunkist Growers				20 000			20 000
Sunsweet Growers Inc.	6 000			13 300	40 700		60 000
Sysco Corporation	9 000	5 000	5 000	5 800			24 800
Taber Creative Group	12 000						12 000
Tandem Diabetes Care Inc.			5 000	5 500			10 500
Tate & Lyle		6 667					6 667
The Beverage Institute		35 823	92 402	61 449			1 89 674
The Sugar Association Inc.					15 600		15 600
The Wonderful Company						21 250	21 250
Triad to Wellness					5 000		5 000
Trovita Health Science			8 250	10 500			18 750
TrovRx Inc.		5 750					5 750
Tzell New England Inc.			6 750				6 750
Unilever Best Foods	76 113	72 295	72 008	43 375	13 000		2 76 791
University of Florida				5 000			5 000
University of Michigan	54 000	92 165					1 46 165
US Foods	5 000	7 000					12 000
US Highbush Blueberry-Padilla CRT					12 400		12 400
USA Rice Federation		5 050					5 050
USDA/FNS/Accounting Division	49 241	1 10 359	29 064	14 455	25 789		2 28 908
Walmart			5 000				5 000
Weber Shandwick				25 500	15 000		40 500
Welch Food					10 000		10 000
Wellington Group Marketing & PR			10 000	15 000			25 000
Wells Blue Bunny				6 500	6 500		13 000
Wells Enterprises Inc.			5 000				5 000
Wild Hive					10 000		10 000
WHO/GSC/GPL				10 000		25 000	35 000
Total	36 88 194	33 16 332	30 80 966	23 90 397	18 62 269	11 19 530	1 54 21 430

AND, Academy of Nutrition and Dietetics; CDR, Constant Default Rate; DPG, Deferred Payment Guarantee.

Second, FOI documents showed large corporate donations to the ANDF from 2011 to 2015, listed in Table 2. Between 2011 and 2014, the Foundation received more than US$2 million each year from corporations, representing approximately a third of its total revenues for that period. In 2015, the corporate funding dropped under US$2 million, but corporate funding still represented more than 62 % of the ANDF’s revenues.

**Table 2:** Academy of Nutrition and Dietetics Foundation revenues 2011–2015

	FY2011	FY2012	FY2013	FY2014	FY2015
Total actual revenue	$3 430 901·94	$3 372 839·52	$3 523 155·51	$3 907 512·09	$3 117 702·75
Breakdown					
Total from members	$926 184·23	$697 719·78	$470 234·92	$1 212 909·01	$881 032·00
Member percentage	27·00 %	20·69 %	13·35 %	31·04 %	28·26 %
Total from CDR-Affiliate – DPG and MIG	$207 699·56	$316 562·32	$329 295·19	$484 034·00	$286 031·14
CDR-Affiliate – DPG and MIG	6·05 %	9·39 %	9·35 %	12·39 %	9·17 %
Corporate					
Grants	$951 610·00	$1 092 574·93	$1 071 190·12	$647 540·45	$487 999·61
Corporate	$1 051 158·15	$566 366·49	$1 204 435·28	$1 366 028·63	$1 365 140·00
Sponsorship	$294 250·00	$699 616·00	$448 000·00	$197 000·00	$97 500·00
Total from corporations	$2 297 018·15	$2 358 557·42	$2 723 625·40	$2 210 569·08	$1 950 639·61
Corporate percentage	66·95 %	69·93 %	77·31 %	56·57 %	62·57 %

CDR, Constant Default Rate; DPG, Deferred Payment Guarantee; MIG, Minimum Income Guarantee.

Third, our findings suggest that ANDF is a means for corporations to reach out to young students and professionals. From 2009 to 2015, corporate contributions to the Foundation were US$15 million. Of these funds, more than US$6 million were transferred to AND members through the distribution of awards, scholarships, research grants, fellowships and other ANDF-led programmes. Of these, US$4·5 million went to an initiative called the ‘Champions Program’, which granted funds to hundreds of non-governmental organisations to support projects ‘promoting healthy eating and active lifestyles for children and their families’ (Academy of Nutrition and Dietetics Foundation Industry Foundation Support Fundraising – Industry Revenue, 2015). At least US$500 000 went to stipends for public nutrition education programmes. Between 2009 and 2012, the General Mills Foundation provided an additional US$2 million directly to the Champions Program and summed a total US$7·5 million in 2015 after 13 years of donations^(33)^.

Lastly, internal AND documents from 2015 to 2016 show that AND invested its funds in the stock of several pharmaceutical companies such as Abbott, Johnson & Johnson, Perrigo Co., Pfizer Inc., Allegra, Merck & Co., and some food and beverage companies such as PepsiCo, Nestlé and J.M. Smucker’s Company.

### Corporate co-opting of nutritionists and dietetic professionals through the Academy of Nutrition and Dietetics/Academy of Nutrition and Dietetics Foundation

The AND certifies US professionals and develops content for continuing professional education as a ‘requirement for’ certification and ‘to build’ knowledge and advance nutritionists’ careers^(34)^. Also, the AND provides a toolkit for individual or organisational members to build their own workshops with continuing professional education credits. Some of the topics of such continuing professional education resources were sponsored or aligned to industry’s interests. For example, ‘Whole Grain Product: Menuing and Getting Kids to Like Them’ was sponsored by General Mills (DM email, 12th June 2015). The ‘Certificate of Training in Childhood and Adolescent Weight Management’ and ‘Changing the Way We Look at Agriculture’ were supported by the National Dairy Council.

The AND also publishes the Journal of the Academy of Nutrition and Dietetics, a monthly peer-reviewed scientific journal. The journal has become a means for the AND to publish its official positions on certain topics^(35)^. For example, the AND has published controversial positions that have been amended over time and appear to be aligned with corporate interests. For instance, in 2017 the AND CEO mentioned to some directors she received an email from the president of the National Dairy Council, concerned about the AND position on vegetarian diets published in the journal^(36)^. The Council’s president indirectly questioned the science behind the public statement mentioning that the National Dairy Council was funding the AND. According to the AND CEO:

[I] Heard an earful yesterday on the phone from Jean as President of Dairy (NDC) about our Vegetarian position paper (six months later?) that has a line in it about dairy and meat. Nothing in the paper says don’t eat dairy or meat or be a vegetarian or vegan but she was saying that Dairy is helping us with funding to elevate the Academy’s science and evidence and it’s so disappointing. I resented the correlation of the sponsorship. (Patricia Babjak, 28th April 2017)

The original position paper on vegetarian diets published in 2015 was retracted at the request of the AND’s Academy Positions Committee, as they ‘became aware of inaccuracies’ and a new version was made public in December 2016, eliminating any mention of specific animal source foods^(36)^. These actions resonate with the commitments to ‘return specific rights and benefits’ to AND/ANDF sponsors, as mentioned in internal documents (JS email, 6th July 2015) but contradict AND’s principle of ‘non-influence’ (point 4, Fig. 2)^(37)^.

### Internal policies and politics favouring corporate ties: the Sponsorship Advisory Task Force

The AND has changed its internal policies to address criticism of its corporate relationships, notably through its SATF. The SATF was established in 2014, following ‘internal and external criticism’ and for ‘enhancing communication and trust among members and the public, and facilitating transparency of process and decisions’^(38)^ (Fig. 2(b)). The SATF was composed of eight AND members and one former director. The advisory group was established to make recommendations and guidelines and review AND’s and ANDF’s policies and practices on corporate-funded programmes and corporate donations.

After the SATF’s produced recommendations, the AND updated its *Guidelines for Corporate Sponsors*. These guidelines require the sponsor’s vision and mission to be aligned with the AND’s vision and mission, that its product portfolio is ‘broadly’ aligned with official AND’s positions and that the sponsorship complies with the Scientific Integrity Principles, principles developed by the International Life Science Institute (ILSI), an industry-funded organisation, in collaboration with the AND in 2015 (Fig. 2)^(39)^. The AND/ANDF also made clear that ‘it does not endorse any company, brand or company products, nor does the AND name or logo appear on any product’^(27)^.

The SATF was not asked to approve the 2014–2015 Kraft-AND partnership. But AND’s strategic communication team commissioned risk assessments ‘with input from an outside source specialised in risk assessments’ noting that some AND members, ‘a vocal, but minor contingent’, would protest the partnership (Kathy Warwick email, 29th March 2015). The BOD weighted the risks and despite the results of the external risk assessment decided to go ahead with the Kraft partnership. AND’s COO conducted a ‘due diligence’ before deciding to ‘accept Kraft as a *National Level Sponsor*’ with an ‘unrestricted gift to *Kids Eat Right*’, recognising that ‘we risk alienating members and/or donors who are not supportive of opportunities to work with big industry’. To accommodate the partnership and justify it Donna Martin, who later became the AND president, had several conversations in August 2014 with the AND’s CFO trying to argue that Kraft Singles could be considered healthy:

We need to change the part that says that the Kraft reduced fat Singles would not meet (USDA) guidelines. Kraft Singles would not meet guidelines, but the reduced fat Singles would (Donna Martin email, AND President, August 29th, 2014).

A majority of AND members, when learning about the Kraft partnership, wrote to the BOD criticising the lack of transparency and their management of the Kraft partnership^(15)^. A member of the BOD talked about the discontent among members and suggested hiding a new agreement being negotiated with Monsanto at that time in order to avoid any further criticism:

‘Dear Board, I think this has moved from educating the members and being appalled that they would believe the New York Times (in relation to their reporting about the Kraft scandal), to an issue of great dissatisfaction with corporate sponsorship, a very sensitive issue and one that we know members are sensitive about, some super sensitive (…) Lets respect and hear them out. They don’t want or deserve a pat on the head. And please, let’s not announce Monsanto any time soon.’ (Aida Miles, ANDF Director, March 2015)

Since the establishment of SATF some food and beverage corporations were no longer listed on the AND sponsorship programme, such as Coca-Cola and Nestlé. Yet, others replaced them such as Abbot Nutrition, Beneo Inc. (food ingredients company), Mead Johnson and others (see Table 1). Some other food and pharmaceutical corporations were still mentioned in AND/ANDF’s annual reports until 2019, either as sponsors or supporters^(40)^.

### Academy of Nutrition and Dietetics allowed companies to purchase rights and benefits

According to AND/ANDF internal communications, AND distinguishes its ‘sponsors’ from its ‘supporters’. Corporate sponsors ‘pay a fee, and in return the Academy provides a right or a benefit’ (JS email, 6th July 2015). Corporate ‘supporters’ provide ‘a charitable contribution with no (explicit) expectation of a commercial return’ (JS email, 6th July 2015). We found several cases when AND has legitimised some corporate positions, which may relate to corporations procuring rights or benefits.

First, we found that the AND established a GMO Task Force in 2017 to work on the AND’s position on GMO. The task force’s report supported the National Academy of Science report, leaning to a critical view of GMO, that would have been a direct criticism of the products of some sponsors, including Monsanto, as discussed by three members of the AND’s BOD (Patricia Babjak, CEO, 8th September 2017). These directors tried to delay the report’s delivery after the September 2017 board meeting, where corporate funding opportunities were to be discussed. Some of the potential sponsors were Abbott, General Mills and Nestlé, and these partners would have had ‘representation in the Board of Directors’ and they ‘will accelerate their organisational nutrition commitments and global public health*’* (Paul Mifsud, CFO, 8th September 2017). Thus, there was the intention to delay any potential criticism about GMO.

Second, we found that in 2016 the AND engaged with the American Society for Nutrition, the Institute of Food Technologists and the International Food Information Council (funded by corporations) to put together a ‘Food and Nutrition Science Solutions Task Force’. They also agreed to write a commentary for a special issue of the *Journal of Public Health*, criticising the NOVA classification of foods, based on their level of processing showing that the consumption of ultra-processed food lead to ill health. At least two of the authors of the proposed papers had ties with the food industry at the time. One was part of the Gerber Foundation and the other was part of the General Mills speaker’s bureau. This group never published the paper, but one of the authors who was an AND member published a criticism of the NOVA classification in a different journal in 2018.

In a final example, in June 2017, the AND CEO emailed other directors that AND had been invited by the US Agriculture Secretary to join the USDA’s new Sodium Awareness Initiative. The goal of this initiative was to reduce sodium in school meals. One director questioned the decision to engage in that initiative, saying that ‘although this is a tremendous HONOR, we do seem to be talking out of both sides of our mouth in regards to sodium’, and recognised that ‘I am well aware that the sodium restrictions in school meals cannot be achieved without industry help, and I am PROUD that we are at the table’. This member pointed out some inconsistencies with AND’s previous actions. In 2015, the AND indeed asked for the US Dietary Guidelines Committee to ‘reconsider the sodium recommendation of 2300 mg/day (considered too low)’, as ‘there is a distinct and growing lack of scientific consensus on making a single sodium consumption recommendation for all Americans, owing to a growing body of research suggesting that the low sodium intake levels recommended by the DGAC are associated with increased mortality for healthy individuals’.

## Discussion

Our findings illustrate different ways in which the AND and its Foundation have interacted and continue to interact with unhealthy commodity corporations in a symbiotic relationship. In a prominent national professional association with high impact on the US food and nutrition practice and US consumers, there are ongoing interactions with food, pharmaceutical and agribusiness corporations. Corporations contribute financially to the AND and its Foundation. The AND/ANDF act as a pro-industry voices in some policy venues, including some of their public positions that clash with AND’s mission to improve health globally.

We have found some internal organisational issues that may compromise the Academy’s mission to improve health. AND leaders have been involved in controversial decisions about corporate relationships and were not removed from their leadership roles despite public disclosure of these relationships. AND has not required its BOD to publicly disclose conflicts of interest, and such disclosures have not been required for membership, as other professional associations have done^(39)^. This research illustrates the extent to which corporate funding enables corporate influence at AND specifically, and across such partnerships more widely. It also suggests this has been normalised, considering the nature of the agreements made and the relationships formed. Although AND has changed some of its internal policies to manage corporate interference and funding, it continues to advance corporate interests in several ways and serves as voice for its corporate sponsors^(41,42)^.

The current AND/ANDF policies and public statements are not sufficient to explain why the AND continues to accept financial contributions of corporations whose products (such as ultra-processed foods and formula milks) are associated with ill health^(43)^. We note however that the association is registered as a trade association, meaning it is an association having common business interests and that can receive contributions from corporations, clashing with its mission statement.

This paper supports previous findings that the AND has implemented minor ‘reforms’ rather than eliminating corporate sponsorship and requiring disclosure of conflicts of interest, with no real perceived commitment to change^(44)^. Our findings suggest that even though some AND members have tried to make AND more transparent, these efforts have not prevailed, and to this day, some basic financial facts and decisions remain secret.

Our results show striking similarities to other cases of institutions captured by corporations, such as the International Life Science Institute and the Global Energy Balance Network, orchestrated by the soft drink industry to promote its commercial agenda in scientific institutions^(45,46)^. For example, in the past, the pharmaceutical industry has been criticised for paying physicians to make income-driven choices leading to the introduction of regulatory frameworks such as the Code of Interaction with Health Care Professionals in the USA or the Physician Payment Sunshine Act to avoid any negative impact of those practices on population health^(47,48)^. There is no such law or rule governing for the interactions between food corporations and nutritionists or dietitians, and they continue to receive grants, scholarships and awards funded by corporations through the ANDF levering their way to continue to influence the profession^(39,49)^.

### Limitations

This study does not include interviews with key actors, which would have provided a detailed narrative of actions and decisions in the AND and ANDF and would have helped contextualise our findings. In addition, some of the information about the AND/ANDF past policies was no longer available online when conducting the study. While this study is not a thorough review of the AND and ANDF policies and procedures around corporate sponsorships and interactions with corporations, it provides insights into several decisions and communications that might have influenced some decisions inside the organisation, pointing to the recent and current internal AND policies, procedures and financial contributions from industry actors. Our analysis was exploratory and not meant to be exhaustive. Hence, the examples presented here only represent a snapshot of the interactions between the AND/ANDF and corporations.

## Conclusion

AND and its Foundation assist the food and beverage, pharmaceuticals and agribusiness industries through their large network of professionals and students, their lax internal policies on corporate partnerships and their topical position papers. The AND/ANDF have been supported financially by these corporations throughout the years despite public criticism and internal organisational changes. With a registration as a trade association, the AND and corporations interact symbiotically. This sets a precedent for close corporate relationships with the food and nutrition profession in the USA, which may negatively affect the public health agenda in the USA and internationally.

## Supplementary material

For supplementary material accompanying this paper visit https://doi.org/10.1017/S1368980022001835. click here to view supplementary material
