# Supplementary material for: The corporate capture of the nutrition profession in the USA: the case of the Academy of Nutrition and Dietetics
Source: Public Health Nutr. 2022 Oct 24;25(12):3568–82. doi: 10.1017/S1368980022001835 (PMC9991767; doi:10.1017/S1368980022001835)
Supplement: Supplementary file 1 [file S1368980022001835sup001.pdf]

Supplementary material. Members of the AND and AND board of directors and their leadership team

Person	AND/AND Foundation position	Description	Link
**Aida Miles, MMSc, RD, LD, FAND**	**Speaker of the House of Delegates 2015-2016**	Professor Miles joined the University of Tennessee, Knoxville, in September 2019 as Clinical Assistant Professor of Nutrition. Aida was the co-director of the Leadership, Education and Training Program in Maternal and Child Health at the University of Minnesota. Aida is also a Paediatric Nutrition Consultant for two clinics that specialize in working with infants and children with feeding disorders, developmental disabilities and Autism Spectrum Disorder. Previously, she was a clinical dietitian at Children's Healthcare of Atlanta and at medical centres in Georgia and Guatemala. She was a member of the AND Board of Directors from 2014-2017 and a Speaker of the House of Delegates.	https://directory.sph.umn.edu/bio/sph-a-z/aida-miles https://nutrition.utk.edu/people/aida-miles/
**Carl Barnes**	**Scholarship Committee Volunteer**	Carl Barnes currently serves as Chief in the US Army in Food Operations for a large medical facility serving more than one million meals per year for patients and patrons. Since 2013, Carl has supported and promoted the mission of the Kids Eat Right campaign to end the childhood obesity epidemic. Carl has also served on the Board of Directors for the Academy of Nutrition and Dietetics Foundation.	https://www.linkedin.com/in/carl-barnes-ms-rd-503b1560/
**David Donnan**	**Board Member (June 2018- to date)**	Donnan is a business leader and consultant to the consumer products, retail and technology industries and a frequent speaker to corporations and groups on topics related to international trade, food and agriculture. He is a partner emeritus with A.T. Kearney, a global management consulting firm, where he ran the global food and beverage practice. During his career, Donnan has managed operating companies, run food plants and consulted with leading global retail and consumer product companies in technology, supply chain strategies and brand growth. He works with major hunger relief organizations such as Feeding America, Global Food Bank Network and Top Box as well as food start-ups in the Chicago area as a mentor. He is a board member of Rubicon Organics, which operates high-technology organic greenhouses in Canada and the U.S. and on the board of Family Farmed, a Midwest non-profit focusing on local, natural and sustainable food systems; and a limited partner with Spiral Sun Ventures that invests in food and agriculture start-ups in the U.S.<br>Donnan is a registered professional engineer and certified management consultant, both in Canada. He is a graduate of the University of Toronto, where he earned a master of business administration degree.	https://www.linkedin.com/in/donnan

**Dianne Polly, JD, RDN, LDN,**	**Speaker of the House of Delegates 2017-2018, chair and member Ethics Committee 2008-2011, Past President and Board Member of TN Dietetic Association (TN Academy of Nutrition) 2005-2019. Foundation board President 2016-2019, Delegate TN (TN Academy of Nutrition and Dietetics)**	Committee Chair and TN chapter President of the Academy of Nutrition and Dietetics. HOD Speaker, Academy of Nutrition and Dietetics. Shelby Country Education Foundation (5y), Metropolitan InterFaith Association (VP), R.E.(22y), Thomason General Hospital (10y)	https://elections.webauthor.com/elections/candidates.cfm?actionxm=Detail&guid=455F0A04-D3D3-EDF4-EB5EA0D0AB938764
**Donna Martin, EdS, RDN, LD, SNS, FAND**	**Academy's 92nd President in 2017-2018**	One-year term on June 1 as the 2017-2018 President of the Academy of Nutrition and Dietetics. Martin, served as the Academy's president-elect during 2016-2017. (1) She is the Director of the School Nutrition Program at Burke County Board of Education for 19 years (2001-to date), which has 4,500 students in five schools, serving breakfast, lunch, after-school snacks and supper. Four of the schools have been acknowledged as HealthierUS School Challenge Gold winners. Martin was the group's electronic communications chair and newsletter editor. She represented the Georgia Dietetic Association in the Academy's House of Delegates and served on the Georgia Department of Education's Task Force on Nutrition Standards for School Nutrition Programs. She served as the Academy's treasurer-elect in 2012-2013 and the Academy's treasurer in 2013-2015. (2)	https://www.eatrightpro.org/media/press-releases/leadership-announcements/martin-becomes-2017-2018-academy-president https://www.linkedin.com/in/donna-martin-eds-rdn-sns-7ab22415/
Elise A. Smith, MA, RD, LD,	**Speaker of the House of Delegates 2013-2014**	She was the director of clinical services for Nutrition Systems, a provider of nutrition consulting and foodservice management.	https://journals.lww.com/nutritiontodayonline/Citation/2012/07000/NUTRITION_Gazette.2.aspx

**Evelyn F. Crayton, RDN, LDN,**	**Academy's 90th President in 2015-2016**	Director, Living Well Associates, Montgomery, AL was president of the Academy of Nutrition and Dietetics. She also is the director and nutrition consultant at Living Well Associates and a professor emeritus at Auburn University, where she worked for more than 35 years. She was a House of Delegates Director on the Academy's Board for two years; served on the House Leadership Team for two years; and was an At-Large member of the Board for three years. A member of the Academy Foundation's Board of Directors since 2013, Crayton served as chair of the Nutrition Education for the Public dietetic practice group and its membership committee and is a past president of the Alabama Dietetic Association, holding offices at all levels of the district association	https://www.linkedin.com/in/evelyn-crayton-edd-rdn-ldn-fand-675aa161/
**Kathleen Niedert, MBA, RD, CSG, FADA, FAND, Treasurer**	**Board of Directors from 2002 to 2005 Member of House of Delegates from 2006 to 2009**	Niedert is the system quality assurance officer for Western Home Services, with responsibility for coaching and training administrators and department heads for operational excellence. Previously, as owner of Omega Health Associates, she was a nutrition consultant to extended care facilities, physicians, substance abuse programs, home health care providers and private industry. Niedert has served as an adjunct faculty member at the University of Northern Iowa, teaching courses in nutrition, human metabolism and food chemistry. A widely published author, she was the editor of the Academy's Nutrition Care of the Older Adult (2016). She is a past president of the Iowa Dietetic Association and past chair of the Academy's Dietetics in Health Care Communities dietetic practice group. Niedert received the Academy's 2012 Marjorie Hulsizer Copher Award, the organization's highest honour, for her service to the Academy and the profession; the 2009 Medallion Award; and the 2001 Award of Excellence in Consultation and Business Practice. Niedert previously served on the Academy's Board of Directors from 2002 to 2005; in the House of Delegates from 2006 to 2009; and as a member of the Commission on Dietetic Registration. Niedert is a graduate of Iowa State University and earned a master's degree in business administration from the University of Northern Iowa.	https://www.eatrightpro.org/leadership/governance/board-of-directors/kathleen-niedert
**Kathy McClusky**	**Head of Sponsorship Task Force Eat right**	Kathy McClusky was the Director of Patient Satisfaction and Consultant at Morrison. She retired in 2015 but still volunteers with the Academy of Nutrition and Dietetics	https://www.foodculinaryprofs.org/docs/Ellection%20and%20Ballot/McClusky%20response.pdf
**Kathy Wilson-Gold, MS, RDN, LD, FAND**	**Chair of AND Foundation 2019-2020, Board Member ANDF 2015-2019, Chair of the Dietitians in Business and Communications DPG**	Past-Chair. She has more than 30 years of experience in business and industry, leading executive teams to deliver innovative and customized business solutions for K-12, C&U and Healthcare. Her career highlights include the Administration on Aging in the Office of Nutrition and Health Promotion Programs, and Campbell Soup Company, Abbott Laboratories and US Foodservice. She has held national and state offices in the Academy, including Chair of the Academy's Foundation Board of Directors, Chair of the Academy's Nominating Committee,	https://eatrightfoundation.org/who-we-are/

	**2013-2014, Chair person of AND Nominating Committee in 2006**	Chair of the Dietitians in Business and Communications dietetic practice group and President of the Texas affiliate. Wilson-Gold serves in her community as a board member of Edmond Mobile Meals and the YMCA.	
**Kay N Wolf**	**Treasurer-Elect of the AND**	Kay N. Wolf became Ohio State's senior vice provost on February 1, 2019, after serving as vice provost for academic policy and faculty resources since November 1, 2014. As senior vice provost, Dr. Wolf oversees the Academic Leaders Forum and other leadership development programs, in addition to various responsibilities within Academic Affairs on a day-to-day basis. Prior to her appointment as vice provost in 2014, Dr. Wolf was director of the health sciences and medical dietetics division in the School of Health and Rehabilitation Sciences, where she holds the rank of professor.<br>A faculty member at Ohio State since 1989, Dr. Wolf studies dietetics education as well as self-efficacy in chronic disease and food security. Her work has been widely published, and it includes an outreach component to vulnerable populations in the Columbus community through Ohio State's Food Innovation Center. Dr. Wolf served three terms on University Senate, serving as co-chair for a subcommittee of the Council on Academic Affairs (CAA). She also served for two years as CAA's chair. She has been honoured with the Academy of Nutrition and Dietetics Service Award and the President and Provost's Award for Distinguished Faculty Service. In addition, she was honoured three times with the School of Allied Medical Professions Service Award. Her teaching has been recognized by the Academy of Nutrition and Dietetics, the College of Medicine and the former School of Allied Medical Professions. Professor Wolf earned her BS in medical dietetics, an MS in food service management and nutrition and a PhD in human resource development from The Ohio State University	https://oaa.osu.edu/wolf-kay
**Kevin L. Sauer, PhD, RDN, LD, FAND, President-Elect**	**Director At-large on the BOD in 2017-2020 Elected President 2021-2022 Several other postions**	Sauer is a professor in the department of food, nutrition, dietetics and health at Kansas State University and co-director of the national Center for Food Safety Research in Child Nutrition Programs. He specializes in administrative dietetics and has previous experience in health care food and nutrition services, school nutrition and university dining. Sauer is an accomplished educator and researcher, receiving the K-State Presidential Award for Excellence in Teaching; the Dawley-Scholer Award for Excellence in Student Development; the Mary Ruth Bedford Distinguished Faculty Award; the Commerce Bank Award for Outstanding Teaching; and the University's Excellence in Engagement Award. He received the Kansas Academy of Nutrition and Dietetics' 2015 Distinguished Dietitian of the Year Award, the 2019 Mentoring Award and the Outstanding Educator Award. A Fellow of the Academy of Nutrition and Dietetics, Sauer's extensive service to the Academy includes past chair of the Commission on Dietetic Registration; past chair of CDR's Examination Panel; and chair of the 2020 CDR RDN/NDTR Entry-Level Practice Audit. He was a director at-large on the Board of Directors in 2017-2020 and	https://www.eatrightpro.org/leadership/governance/board-of-directors/Kevin-l-sauer

		served on the Council on Future Practice; House of Delegates Evolution Design Team; Council on Research; Nutrition and Dietetics Educators and Preceptors Council; Research Priorities and Development Task Force; Code of Ethics Revision Task Force; and the Academy Foundation's Healthy and Sustainable Food Systems collaborative. Sauer will serve as the Academy's president in 2021-2022. He attended Dodge City Community College and received undergraduate, master's and doctoral degrees from Kansas State University.	
**Linda T. Farr, RDN, CSOWM, LD, FAND**	**She was Speaker of the House of Delegates in 2016-2017, and it is the Academy's President-elect in 2019-2020**	Farr is the owner of Nutrition Associates of San Antonio, doing business as Nutritious Table. She is a board-certified specialist in obesity and weight management and provides personalized medical nutrition therapy to teens and adults and serves as a source for media interviews and participates in public speaking engagements. A former clinical dietitian and hospital foodservice director, Farr has more than 35 years of experience in medical, surgical, mental health, physical rehabilitation and private practice settings. She is a Medicare and insurance provider and a preceptor for several dietetic internships, focusing on reimbursement and business practices. In the Academy's House of Delegates, Farr served on the House Leadership Team and as a two-term Texas affiliate delegate. She was Speaker of the House of Delegates in 2016-2017.<br><br>She is a founding member of the Weight Management dietetic practice group. Farr served as the Academy's President-elect in 2019-2020 and is a past president of the Academy's Texas, San Antonio and Dallas affiliates. Farr was named a 2005 San Antonio Healthcare Hero by the San Antonio Business Journal and was the Texas Academy's 2011 Distinguished Dietitian of the Year. Farr was appointed to the San Antonio Mayor's Fitness Council and served as a volunteer for the city's Por Vida! healthy restaurant initiative. She was instrumental in developing nutrition guidelines for diabetes-friendly restaurant choices and healthful vending machine criteria earning praise from the federal government. Farr is a graduate of Iowa State University	https://www.eatrightpro.org/leadership/governance/board-of-directors/Linda-t-farr-rdn-csowm-ld-fand
**Lucille Beseler, MS, RDN, LDN, CDE, FAND, Academy's 91st President in 2016-2017**	**Academy's 91st President in 2016-2017 and 2015- 2016/ Elected to the Board of Directors for a three year term from 2011-2014**	Owner of the Family Nutrition Center of South Florida. She opened FNCSF 29 years ago and provides comprehensive nutrition services to consumers, and corporations. Currently, Beseler serves on a number of scientific advisory boards for the following companies: Mead Johnson, Kate Farms, and Advanced Micronutrition. Lucille Beseler served on the Academy Board of Directors for three years prior to her being elected President in 2016-2017. Beseler is a past chair of the Academy's Political Action Committee and a member of its Childhood Obesity Coalition. She was elected as President of the Florida Academy of Nutrition and Dietetics after serving nine years as chair of the Florida Dietetics Nutrition Practice Council – the state licensing board for dietitians and nutritionists – and received a public service award from the Florida Academy of Nutrition and Dietetics.	https://www.linkedin.com/in/lucille-beseler-ms-rdn-cde-fand-52323723/

**Margaret P. Garner, MS, RDN, LD, CIC, FAND**	**Chair of AND Foundation 2020- 2021, Past Treasurer 2017-2018, Treasurer 20016-2017**	Garner is an associate professor of family medicine at the University of Alabama and the executive director of the student health center, where she also serves as the director of the Department of Health Promotion and Wellness. Chair of AND Foundation. Garner helped establish and was the first chair of the Alabama Food and Nutrition Exposition, a unique partnership of the Alabama Dietetic Association, Alabama Dietary Managers Association and Alabama School Nutrition Association. In 2011, she received the Academy's highest honour, the Marjorie Hulsizer Copher Award. Garner has served in many leadership roles in the Academy, including as a member of the Board of Directors and Commission on Dietetic Registration. She was a member and chair of the Academy's Legislative and Public Policy Committee, Political Action Committee, Coding and Coverage Committee, Council on Education and Strategic Planning Task Force. Garner is a past president of the Tuscaloosa District Dietetic Association and the Alabama Dietetic Association.	https://eatrightfoundation.org/who-we-are/
**Mary Beth Whalen**	**Director of Academy of Nutrition and Dietetics Foundation 2014- 2015**	Chief operating officer AND Executive Director since Aug 2014. She provides leadership for strategic communications, marketing, corporate relations, business development and philanthropy for the largest organization of food and nutrition professionals in the world, representing over 100,000 Registered Dietitian Nutritionists. Serves as Executive Director of the Academy of Nutrition and Dietetics Foundation, the organization's $30 million 501(c)3. North-western Uni BA in Communications (1983)	https://www.linkedin.com/in/mary-beth-whalen-a8b5769
**Mary Beth Whalen**	**Chief Operation Officer 2014- present**	Chief Operations Officer of the AND from June 2014 to present. Serves as Executive Director of the Academy of Nutrition and Dietetics Foundation, the organization's $30 million 501(c)3.	https://www.linkedin.com/in/mary-beth-whalen-a8b5769
**Mary Pat Raimondi**	**VP Strategic Policy and Partnership at the Academy of Nutrition and Dietetics from June 2020 to Jul 2017**	Mary Pat saved as the Program Director of University of Minnesota from 2008 to 2010, and was Partner of the Milestone Group, a group of restaurants, from January 1997 to July 2019,and before he was the Senior Product Manager at Novartis for 10 years.	https://www.linkedin.com/in/mary-pat-raimondi-81ba544/ https://themilestone.group/
**Mary Russell, MS, RDN, LDN, FAND,**	**Academy's 93rd President in 2018-2019.**	Mary Russell has been a member of the AND for a long time. She was a treasurer-elect, treasurer and immediate past treasurer from Jun 2010 to May 2015. She was chair of the Ethics Committee for 1 year and member for two (from June 2013 to may 2016). Chair- Elect, Chair and immediate past chair from Jun 2001 to may 2004.	https://www.linkedin.com/in/mary-russell-ms-rdn-ldn-fand-faspen-9131249/

**Milton Stokes, PhD, MPH, RD, FAND**	**House of Delegates Director (3 year position) 2017-2018, 2018-2019 and 2019-2020**	Global Lead, Public Affairs and Issues Management at Bayer Crop (2020'2021), and previously Director of Global Health and Nutrition Outreach at Bayer Corp (2018-2020) and Monsanto (2014-2018) owned by Bayer Corp now. He is an active member of the Academy of Nutrition and Dietetic. He served in a variety of volunteer, appointed, and elected leadership positions, including National Media Spokesperson in the New York City media market where he featured in Cooking Light, Fitness, Self, Men's Health, AOL, The Washington Post, Ladies Home Journal. He has written freelance articles for Environmental Nutrition, Today's Dietitian, Family Doctor, Men's Health, WeightWatchers.com, NY Daily News, and others.<br><br>He is a clinical nutrition specialist and has worked on special projects and per diem with Unidine in New England. He has served in staff and management positions along the way as well as preceptor dietetic interns and students from several colleges and programs. His graduate degree is in Health Education from Hunter College, and his clinical training was conducted at Yale-New Haven Hospital. My PhD is in Health Communication from the University of Connecticut.	https://www.linkedin.com/in/Milton stokes
**Patricia M Babjak**	**AND and ANDF CEO since 2009**	She has served as the Academy's CEO since 2009. She oversees the Academy's Headquarters Team, including 170 employees and a $70 million annual budget. Babjak is also CEO of the Academy's Foundation, the organization's philanthropic arm and the only charitable organization devoted exclusively to promoting nutrition and dietetics. Babjak joined the Academy in 1975 and became executive director of the Commission on Dietetic Registration in 1978, serving until 1998 when she became executive vice president for Strategic Management and Governance. As executive vice president, she was responsible for overseeing the development and implementation of the Academy's strategic plan and for developing mechanisms to measure the Academy's progress toward goals and objectives. Babjak facilitated the reorganization of the Academy's governance structure, including new roles for the Board of Directors and House of Delegates and the transformation of the Academy's Nominating Committee into a force for leadership, development and diversity. Babjak served as the Academy's interim chief executive officer in 1997. In 2004, in recognition of her service to the Academy and to the nutrition and dietetics profession, Babjak was awarded honorary Academy membership. Babjak served on the Advisory Committee of the Harvard University Leadership Institute and the Pew Health Professions Commission on Educating Health Care Workforce Task Force. She was nationally elected by other professions to chair the National Commission for Certifying Agencies and serves on the Leadership Council for the National Organization for Competency Assurance.	https://www.eatrightpro.org/leadership/governance/board-of-directors/patricia-m-babjak-gslis

		Babjak is a graduate of the University of Illinois at Chicago and earned a master's degree in library and information science from Dominican University in Illinois	
**Paul Mifsud**	**Chief Financial Officer of the AND/ANDF 2000 to date**	Responsible for the financial planning, analysis and administrative management of a 75,000 member association. This includes/has included; Financial Planning and Analysis, Forecasting, Accounting Operations, Budgets, Contract Management, Bank Relations, Risk Management, Capital Management, Tax, I/T, MIS, Purchasing, Publications, Legal, Human Resources, Meeting Services and Office Service. From 1984 t0 2000 he was the CFO of also the CFO of Ameritech Consumer Business Unit (now AT&T).	
**Terri J. Raymond, MA, RDN, CD, FAND**	**2019-2020 President, and Head of ANDF**	Raymond is the president and owner of Dietitian Consulting Service LLC. Previously, she worked at Food Management Corporation in positions ranging from director of clinical consultation to regional manager, vice president and president. Raymond served as the Academy's 2019-2020 president. Her service to the Academy includes being a member of the Finance Committee; chair of the Professional Development Committee; member of the Competency Assurance Panel and Appeals Panel of the Commission on Dietetic Registration; and member of the Leadership Institute Oversight Task Force. Raymond is a past chair of the Board of the Academy's Foundation and served on the Foundation's Board for five years. She is a past president of the Washington State Academy of Nutrition and Dietetics; received the state's Distinguished Service Award and represented Washington State in the Academy's House of Delegates. Raymond is a past chair and member of the Dietitians in Business and Communications dietetic practice group's executive board. She received the DPG's Founder's Award and the Dietitians in Health Care Communities dietetic practice group's Abbott Leadership Award. Raymond is a graduate of Washington State University and earned a master's degree from Antioch University.	https://www.eatrightpro.org/leadership/governance/board-of-directors/terri-j-raymond

**Sonja Connor, MS, RDN, LD**	**89th President of the Academy of Nutrition and Dietetics form 2014 to 2015**	Sonja is a research associate professor in the Division of Endocrinology, Diabetes and Clinical Nutrition, Department of Medicine, School of Medicine, Oregon Health & Science University Oregon Health Sciences University. At OHSU, Connor is the director of the nutrition elective for medical students, as well as the developer of the nutrition course for physician assistant students. Connor previously was chair-elect of the Academy Foundation's Board of Directors; chaired the Foundation's Scholarship Committee; and is a former member of the Academy's Nominating Committee. In 2005-2006, she was the Speaker of the Academy's House of Delegates. She served on the Academy's Strategic Planning Initiative Task Force and was a research delegate to the Academy's Council on Professional Issues. She is currently the director of the Seafood Nutrition Partnership a nonprofit working to raise awareness in the US about the 'essential nutritional benefits of eating seafood.'	https://jandonline.org/article/S2212-2672(14)00424-9/fulltext https://www.prnewswire.com/news-releases/registered-dietitian-nutritionist-sonja-connor-becomes-2014-2015-president-of-academy-of-nutrition-and-dietetics-261536241.html https://www.seafoodnutrition.org/
**Glenna McCollum, MPH, RDN**	**88th President of AND 2013-2014 Speaker House of Delegates (June 2010 to May 2011)**	Glena was a clinical dietitian in the Mesa Lutheran Hospital from 1985 to 1987, then she was the Dean Assistant and Director of Nutrition at Central Arizona College from 1990 to 2007. She was also the CEO of the Chandler Education Foundation for 13 years, and an advisor for Phyco BioSciences, Inc.	https://www.linkedin.com/in/dr-glenna-mccollum-mph-rdn-0599891/
